# Hyperhomocysteinemia is an independent risk factor of atherosclerosis in patients with metabolic syndrome

**DOI:** 10.1186/s13098-019-0484-0

**Published:** 2019-10-26

**Authors:** Giuseppina Piazzolla, Mafalda Candigliota, Margherita Fanelli, Anna Castrovilli, Elsa Berardi, Gianfranco Antonica, Stefano Battaglia, Vincenzo Solfrizzi, Carlo Sabbà, Cosimo Tortorella

**Affiliations:** Interdisciplinary Department of Medicine, University of Bari School of Medicine, Piazza G. Cesare 11, 70124 Bari, Italy

**Keywords:** Homocysteine, Metabolic syndrome, IMT, Carotid stenosis, C-peptide, Folic acid, Pack-years

## Abstract

**Background:**

Metabolic syndrome (MetS) is a clinical condition potentially promoting the development of atherosclerotic disease. To date, the clinical impact of elevated serum homocysteine (Hcy) levels in MetS is still under discussion. The aim of this cross sectional study was to evaluate the relationship between MetS and hyperhomocysteinemia and the potential role of Hcy in the pathogenesis of atherosclerotic complications of MetS.

**Methods:**

We recruited 300 outpatients with MetS. All patients underwent a medical history collection, physical examination, blood sampling and carotid ultrasound echo-color Doppler. According to Hcy levels, MetS patients were divided into two groups: “normal” (< 10.7 μmol/l; n = 140, group 1) and “high” Hcy (≥ 10.7 μmol/l; n = 160, group 2). Comparisons between groups were made by Student’s t-test or Chi-square test. The effects of potential covariates on group differences were evaluated by general linear models. The relationships between continuous variables were assessed by simple or multiple correlation and by linear regression. Multiple regression models were built to evaluate the effects of Hcy, together with other potential risk factors, on carotid atherosclerosis.

**Results:**

Patients with high Hcy were predominantly male and slightly older than group 1 patients. Smokers and non-smokers exhibited similar Hcy levels, nor was a statistical relationship between pack-years and Hcy observed. Group 2 showed lower levels of folic acid, vitamin D, high density lipoprotein (HDL)-cholesterol and glomerular filtration rate (e-GFR) than group 1, but higher levels of C-peptide, uric acid and triglycerides. In all patients, Hcy was positively correlated with C-peptide and uric acid and negatively with folic acid and e-GFR. Intima-media thickness (IMT) and carotid stenosis degree were significantly higher in patients with high Hcy and a positive relationship between Hcy and both IMT and carotid stenosis was detected in all patients. Finally, Hcy atherogenic effects were independent of other well-known atherosclerosis risk factors.

**Conclusions:**

Our results highlight a link between MetS and hyperhomocysteinemia and a direct effect of Hcy on atherogenic process during MetS. Early correction of folic acid levels may contribute to prevent cardiovascular complications in MetS patients.

## Background

Cardiovascular diseases are still the main cause of mortality worldwide. Early identification, prevention and treatment of the main cardiovascular risk (CVR) factors are undoubtedly priority goals to be achieved. In this context, the metabolic syndrome (MetS) and increased serum homocysteine (Hcy) levels have been described as independent risk factors for cardiovascular diseases [1, 2].

Epidemiological and clinical data support an important link between MetS and hyperhomocysteinemia, and some studies have even suggested that increased serum levels of this amino acid may be an additional constituent of MetS [3]. However, the exact nature of this relationship remains unclear. In all these clinical conditions the complex interaction of genetic and environmental factors, among which a sedentary lifestyle and overnutrition play a determining role, contributes to the onset and persistence of a low-grade systemic inflammation. This pro-inflammatory state, in turn, seems to affect the incidence of systemic complications, first of all atherosclerotic disease [4].

The MetS is a common condition whose prevalence is rising to epidemic proportions, having a significant impact on public health and the global economy [5, 6]. Far from being a classic disease in itself, it is rather defined as a cluster of metabolic disorders (i.e. insulin resistance, central obesity, dyslipidemia, hypertension, endothelial dysfunction) whose coexistence in the same individual predisposes to cardiovascular (CV) events, type-2 diabetes and nonalcoholic fatty liver disease [5].

Since 1998, scientific organizations have proposed several closely related but individual definitions of the syndrome, using diagnostic criteria based on different cut-off points of risk values for waist circumference, blood pressure and serum levels of glucose, triglycerides and high density lipoprotein (HDL)-cholesterol. Regardless of which cluster of criteria was adopted, the common purpose has been to make an early identification of high-risk patients and potential CV complications. In 2009, the so-called “harmonizing definition” of MetS assigned equivalent levels of importance to all of its components, thus reconciling all previous definitions [7].

Homocysteine is a dietary amino acid produced by the breakdown of methionine [8] whose metabolic pathway involves several enzymes. Circulating Hcy is, in fact, maintained at relatively low levels both by vitamin B12/folate-dependent enzymatic conversion to methionine and via a trans-sulfuration pathway forming cysteine [9]. Genetic variability of one of these enzymatic activities [10, 11], as well as folate or vitamin B12 deficiencies [12], may induce increased levels of cellular and serum Hcy, considered to be toxic to cells [8]. Other conditions associated with hyperomocysteinemia include age, smoking and male sex.

The primary purpose of the study was to evaluate the controversial relationship between Hcy and Mets. At the same time, the possible role of Hcy in the pathogenesis of atherosclerotic complications of the MetS was investigated via color-doppler ultrasonography of carotid vessels, a common method that has proven to be both reliable and inexpensive.

## Patients and methods

### Study population

In this cross sectional study, 300 consecutive patients with MetS (182 males and 118 females; mean age 63 years, range 26–86 years) attending the Metabolic Disorders Outpatients Clinic of the Department of Internal Medicine at the University Hospital of Bari, were enrolled from December 2017 to November 2018. MetS was diagnosed, according to the “harmonizing definition” of the syndrome [7], when at least three of the following criteria were present: (1) waist circumference ≥ 94 cm in European men or ≥ 80 cm in European women; (2) fasting glucose > 100 mg/dl or ongoing therapy for elevated glucose levels; (3) triglycerides ≥ 150 mg/dl or specific treatment for this lipid abnormality; (4) HDL < 40 mg/dl in men or < 50 mg/dl in women or specific treatment for this lipid abnormality; (5) systolic blood pressure ≥ 130 mmHg and/or diastolic blood pressure ≥ 85 mmHg or ongoing therapy for hypertension. Exclusion criteria were any kind of cancer within less than 5 years prior to the study, infections and systemic corticosteroid treatment within 4 weeks prior to the study. All patients underwent a general examination, including the following measures: height, weight, Body Mass Index (BMI), waist circumference, arterial pressure; venous sampling for routine analysis, including serum Hcy, uric acid, vitamin B12, folic acid, 25-OH vitamin D, C-peptide and glycated haemoglobin (HbA1c), after 12-h fasting period; and instrumental tests including echo-color Doppler of the supra-aortic vessels.

Patients were subdivided into two groups according to the detection of “normal” (< 10.7 µM/l; group 1) or “high” (≥ 10.7 µM/l; group 2) serum Hcy values.

All biochemical measurements were centralized and performed in the ISO 9001 certified laboratories of the University Hospital of Bari. Hcy and uric acid were determined by the URCA method on the Dimension Vista System (Siemens Healthcare Diagnostic Products GmbH, Marburg, Germany); vitamin B12 and folic acid were assessed by chemiluminescent immunoassay (CLIA) on the ADVIA Centaur System (Siemens); C-peptide and 25-OH vitamin D were determined by CLIA on the LIAISON analyzer (DiaSorin Inc, Stillwater, MN, USA); HbA1c was assessed in human whole blood using ion-exchange high-performance liquid chromatography (HPLC) on the Bio-Rad Variant II Hemoglobin A1c Program (BIO-RAD Laboratories Srl, Milan, Italy).

Echo-color Doppler, always carried out by the same two operators, allowed us to measure the Intima Media Thickness (IMT) and to calculate the degree of carotid stenosis. All the examinations were performed with high resolution B-mode ultrasonography (Esaote Mylab Twice) using a 7–12 MHz vascular transducer in multiple projections (longitudinal and transverse scans) to optimize detection of the parameters. The evaluation of the carotid arteries included three modalities: B-mode, in order to get images in grey scale for the measurement of IMT 1 cm proximal to the carotid bulb; color doppler and spectral analysis, in order to obtain the blood flow velocity. Combining all the information collected through these three different approaches it was possible to study the degree of stenosis and to classify its severity in categories according to ultrasound and doppler flowmetric values. In particular, the NASCET (North American Symptomatic Carotid Endarterectomy Trial) method was used to quantify the degree of stenosis. The IMT, defined as the distance between the intimal-luminal interface and the medial-adventitial interface, was considered pathological if greater than 0.9 mm.

The HOMA (Homeostasis Model Assessment) Index was calculated as (fasting insulin × fasting glucose)/405. A pack-year was defined as 20 cigarettes smoked every day for 1 year.

The study was approved by the Clinical Investigation Ethics Committee of the University of Bari Medical Center (Ethical approval number: PZZ/SM_BPCO/2017), and all patients gave written informed consent to take part.

### Statistical analysis

Comparisons between groups were made by Student’s t-test and Chi-square test for continuous and categorical variables, respectively. As age, gender and smoking habit were significantly different between patients with normal or high Hcy, the analysis of covariance was carried out by general linear models to correct further differences between the two groups by the effects of these covariates on Hcy.

The relationship between independent and dependent continuous variables was assessed by linear regression. The effects of Hcy, together with age, pack-years, sex and other potential atherosclerosis risk factors, on IMT and carotid stenosis were evaluated by multiple regression models. In particular, a forward stepwise selection based on Akaike information criterion (AIC) was performed after an automatic smoothing of outliers and a correction of missing values.

Any other association among variables was evaluated by simple or multiple correlation. In the latter context, partial Pearson correlation coefficients were calculated for each pair of variables when the effect of remaining variables had been removed.

Statistical analysis was performed with SPSS version 23.

## Results

The baseline characteristics of our study population are summarized in Table 1. More than 50% of total patients exhibited higher than normal levels of serum Hcy.

**Table 1:** Baseline characteristics of the total study population

	Patients with normal homocysteine (group 1; n = 140)	Patients with high homocysteine (group 2; n = 160)	Statistical analysis	Significance (p)
Age (years)	61.4 ± 0.9	64.1 ± 1.0	t = − 2.015	p = 0.045
Gender, male [n (%)]	67 (47.9%)	115 (71.9%)	χ^2^ = 18.050	p < 0.001
Smokers [n (%)]	58/137 (42.3%)	85/155 (54.8%)	χ^2^ = 4.549	p = 0.033
Never smokers [n (%)]	79/137 (57.7%)	70/155 (45.2%)		
Pack-years (n)	11.6 ± 1.9	19.4 ± 2.5	t = − 2.464	p = 0.014
^a^				
Waist circumference (cm)	104.1 ± 1.1	105.3 ± 1.1	F = 0.62	p = 0.432
BMI (kg/m^2^)	29.7 ± 0.5	30.0 ± 0.5	F = 0.20	p = 0.659
SBP (mmHg)	129.2 ± 1.2	130.9 ± 1.2	F = 0.96	p = 0.328
DBP (mmHg)	79.3 ± 0.8	79.0 ± 0.8	F = 0.04	p = 0.837
MetS criteria (n)				
3	60 (42.9%)	60 (37.5%)	χ^2^ = 6.723	p = 0.035
4	63 (45.0%)	62 (38.7%)		
5	17 (12.1%)	38 (23.8%)		
Fasting glucose (mg/dl)	113.0 ± 3.4	116.1 ± 3.4	F = 0.41	p = 0.521
HbA1c (mmol/mol)	45.6 ± 1.2	43.9 ± 1.2	F = 0.97	p = 0.324
C-peptide (ng/ml)	2.0 ± 0.1	2.4 ± 0.1	F = 7.10	p = 0.008
Insulin (microUI/ml)	11.4 ± 0.7	12.0 ± 0.7	F = 0.30	p = 0.587
HOMA Index	3.1 ± 0.2	3.4 ± 0.2	F = 0.79	p = 0.376
Vitamin D (ng/ml)	23.5 ± 0.9	21.0 ± 0.9	F = 4.06	p = 0.045
Folic acid (ng/ml)	9.0 ± 0.4	6.7 ± 0.4	F = 15.76	p < 0.001
Vitamin B12 (pg/ml)	414.6 ± 29.6	401.0 ± 28.8	F = 0.11	p = 0.744
e-GFR (ml/min)	90.4 ± 1.2	81.3 ± 1.2	F = 26.85	p < 0.001
Creatinine (mg/dl)	0.78 ± 0.03	0.93 ± 0.03	F = 15.27	p < 0.001
BUN (mg/dl)	37.0 ± 1.2	44.8 ± 1.2	F = 19.11	p < 0.001
Uric acid (mg/dl)	4.5 ± 0.1	5.0 ± 0.1	F = 12.32	p = 0.001
Cholesterol (mg/dl)	163.4 ± 3.1	165.0 ± 3.1	F = 0.14	p = 0.705
LDL-cholesterol (mg/dl)	85.9 ± 2.9	87.7 ± 2.9	F = 0.19	p = 0.662
HDL-cholesterol (mg/dl)	53.6 ± 1.1	49.2 ± 1.1	F = 4.10	p = 0.044
Triglycerides (mg/dl)	122.1 ± 6.0	148.8 ± 5.9	F = 9.50	p = 0.002
Carotid atherosclerosis^b^ [n (%)]	111/133 (83.5%)	139/154 (90.3%)	χ^2^ = 2.939	p = 0.086
Carotid stenosis (%)	19.1 ± 1.6	23.5 ± 1.4	F = 4.19	p = 0.042
IMT (mm)	1.02 ± 0.01	1.10 ± 0.02	F = 4.04	p = 0.046

Data are presented as mean ± SE or as frequency and percentage. Evaluation of group differences was done with Student’s t test and the Chi-square test for continuous and categorical variables, respectively

A pack-year is defined as 20 cigarettes smoked every day for 1 year

*BMI* Body Mass Index, *SBP* systolic blood pressure, *DBP* diastolic blood pressure, *HbA1c* glycated haemoglobin, *HOMA* (Homeostasis Model Assessment) Index: fasting glucose × fasting insulin/405, *e-GFR* glomerular filtration rate, *BUN* blood urea nitrogen, *LDL* low density lipoprotein, *HDL* high density lipoprotein, *IMT* Intima Media Thickness

^a^Age, sex and smoking were used as covariates to perform further evaluations by general linear models. Therefore, from here, continuous variable values are expressed as corrected means, as estimated by the model

^b^Patients showing an IMT greater than 0.9 mm and/or a carotid stenosis

**Fig. 1:**
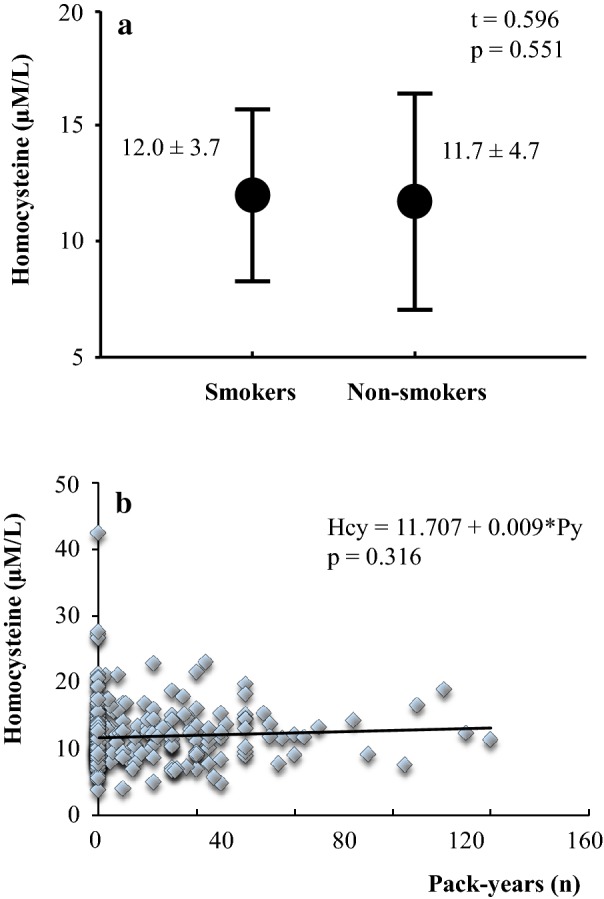
**a** Hcy levels in smoker and non-smoker patients with MetS. Values are expressed as mean ± SD. **b** Relation between pack-years and Hcy in all MetS patients. Each symbol identifies a single individual

The subset of patients with high Hcy (group 2) consisted mainly of males. This is no coincidence, as the amino acid levels appeared to be significantly higher in males than in females (mean ± SD: 12.7 ± 4.7 vs 10.5 ± 3.9, respectively; t = 3.780, p < 0.001). In addition, the patients in group 2 were slightly but significantly older than those in group 1, in line with known epidemiological data. The number of pack-years was higher in patients with high Hcy, and the percentage of smokers was significantly different between the two groups (Table 1). Nevertheless, Hcy levels were not different between smoker and non smoker patients (Fig. 1a) and no relationship was detected in the whole population between pack-years and serum Hcy levels (Fig. 1b).

Given the role of sex, age and smoking on Hcy metabolism, these variables were used as covariates to correct the additional differences detected between patients with normal or high Hcy by their potential effects. Accordingly, for all other continuous variables, corrected means, as estimated by the analysis of covariance model, are reported in Table 1.

All patients showed high absolute values of waist circumference, BMI and HOMA Index but no differences between the two groups were observed as regards these or other clinical variables (Table 1). As concerns the blood chemistry data, the group of patients with high Hcy was characterized by higher values of C-peptide, triglycerides and uric acid and lower concentrations of HDL-cholesterol, folates and vitamin D compared to the other group (Table 1). Interestingly, half of these variables appeared to be related to serum Hcy. A multiple correlation analysis between Hcy and all these variables, in fact, revealed a negative correlation between Hcy and folate as well as a positive association between Hcy and C-peptide or uric acid (Table 2).

**Table 2:** Partial correlation coefficients between Hcy and metabolic variables in MetS patients

	C-peptide	Triglycerides	Uric acid	HDL-cholesterol	Folic acid	Vitamin D
Homocysteine	r = 0.274 p < 0.001	r = − 0.047 p = 0.483	r = 0.182 p = 0.006	r = − 0.410 p = 0.540	r = − 0.287 p < 0.001	r = − 0.050 p = 0.452

The analysis of kidney function tests highlighted higher levels of (blood urea nitrogen) BUN and creatinine, and lower values of glomerular filtration rate (e-GFR) in the subset of patients with high Hcy (Table 1). In addition, examining MetS individuals as a whole, a negative correlation between Hcy and e-GFR (r = − 0.441; p < 0.001) and, concordantly, a positive correlation of the amino acid concentration with both BUN (r = 0.557; p < 0.001) and creatinine (r = 0.624; p < 0.001), were observed.

**Fig. 2:**
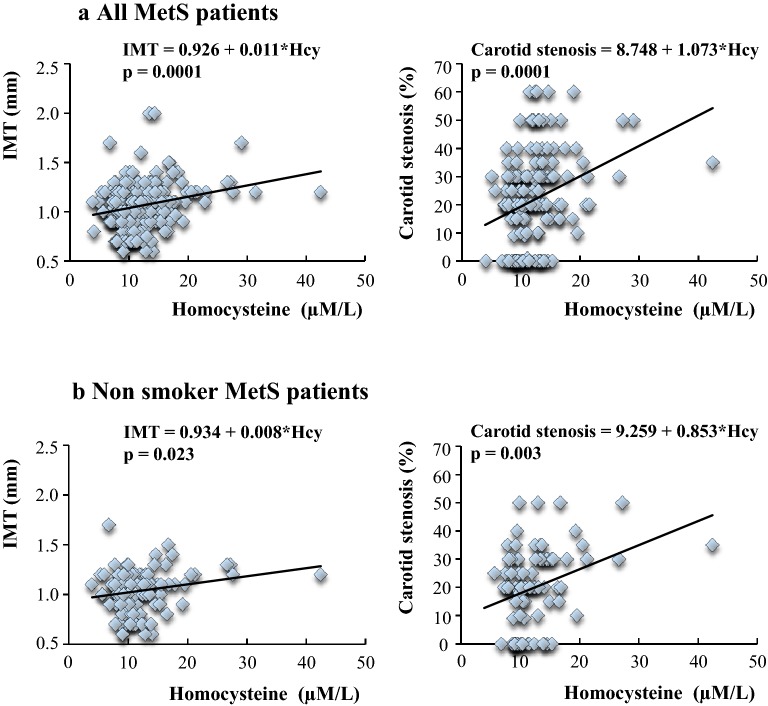
Effects of Hcy on IMT and carotid stenosis degree in all (**a**) and in non smoker (**b**) MetS patients. Each symbol identifies a single individual

Finally, we evaluated the possible relationship between Hcy and atherosclerosis in the course of MetS. In this respect, it should be outlined that more than 80% of our patients showed signs of carotid atherosclerosis, defined as an IMT greater than 0.9 mm and/or a carotid stenosis. This finding was independent of the levels of Hcy, the percentage of patients with ultrasound signs of atherosclerosis being not significantly different in the subset with high compared with normal amino acid serum concentrations (Table 1). However, when the degree of atherosclerosis was quantified, in terms of either IMT or carotid stenosis, values were significantly greater in group 2 (Table 1). Consistent with these data, a significant effect of Hcy levels on IMT or carotid stenosis degree was detected in the whole population of MetS patients, as indicated by the positive relation between Hcy and either of these atherosclerosis clinical signs (Fig. 2a). Accordingly, we deemed it necessary to assess whether the promoting action of Hcy on atherosclerotic disease might be due to other confounding atherosclerosis risk factors, including, in particular, a smoking habit. This did not seem to be the case as a significant positive relation between Hcy and IMT or carotid stenosis was observed even when considering only non smoker MetS patients (Fig. 2b).

Furthermore, multiple regression analysis demonstrated that Hcy, pack-years and age, but not sex, systolic blood pressure, e-GFR, low density lipoprotein (LDL)-cholesterol or triglycerides, were able to affect IMT (Final Model: IMT = 0.466 + 0.07 * Age + 0.02 * Py + 0.008 * Hcy; p < 0.001). Similar results were detected when the carotid stenosis degree was evaluated (Final Model: Carotid stenosis = − 24.554 + 0.595 * Age + 0.22 * Py + 0.811 * Hcy; p < 0.001).

The analysis of the atherogenic effect of uric acid was not among the purposes of the study. Nevertheless, the observation that uricemia did not relate either with IMT (IMT = 1.04 + 0.005 * Uric acid; p = 0.614) or with the percentage of carotid stenosis (Carotid stenosis = 15.75 + 1.22 * Uric acid; p = 0.173), although incidental, is worthy of mention.

## Discussion

An increasing number of studies over the last 20 years has underlined the role of Hcy as a potential risk factor for CV diseases, venous thrombosis, vascular complications on atherosclerotic basis, systemic disorders, cognitive decline [1, 13–17]. It has also been reported that its effects on atherogenic processes are particularly relevant in diabetic patients [18–20]. To date, despite the ample volume of literature available, the clinical impact of elevated serum Hcy levels is still not clear, and whether Hcy is a mediator, a biomarker or just an epiphenomenon of cardiovascular and/or neurological diseases is still under discussion [9].

In this context, the MetS, a clinical condition associated with a high mortality for CV events and for all causes, appears to be closely linked to homocysteinemia but the expert opinions on the exact nature of this relationship are controversial [21, 22]. In fact, the data provided by studies in both the general population and specific subgroups of individuals, such as adolescents, obese patients, or those with previous CV disease [19, 21, 23, 24], are conflicting.

The present study outlines a significant association between the MetS and hyperhomocysteinemia. Contrary to the prevalence of hyperhomocysteinemia ranging around 5–10% in the adult general population, with peaks of 30% in the elderly [25], a very high percentage of MetS patients included in the study (53.3%) showed higher than normal Hcy levels. In line with the epidemiological data for the general population, a large proportion of these patients was male (71.9%), likely as a result of the gender-dependent production of Hcy being higher in males than females [26]. The comparison of MetS patients with normal or elevated serum Hcy levels highlighted the fact that the group with high Hcy exhibited significantly higher triglycerides and lower HDL-cholesterol values, two parameters that contribute directly to the diagnosis of MetS. Interestingly, the same subset of patients also showed significantly higher levels of uric acid, a molecule that, like Hcy, is counted among the emerging CVR factors, a serum increase being considered a potential additional diagnostic criterion of the MetS.

A particularly interesting point emerged from the analysis of the smoking habit variable, as cigarette smoke is described to induce both insulin resistance [27] and Hcy production [8]. The group with high Hcy displayed higher values of pack-years and a greater percentage of smoking individuals (including both ex-smokers and current smokers) as compared with the other subset. Nevertheless, such an important CVR factor did not appear to affect the amino acid serum levels in patients with MetS, as serum Hcy was similar in smokers and non-smokers and, above all, no significant relation between pack-years and Hcy levels was observed in the overall study population. According to these data, we conclude that a smoking habit is not a discriminating factor between MetS patients with high or normal homocysteinemia and, therefore, it should not significantly influence Hcy-mediated cardiovascular effects in our patients.

In this regard, it is worth noting that MetS patients with high Hcy exhibited significantly greater C-peptide values compared with the other group. As known, C-peptide levels depend only on insulin production, are not affected by insulin replacement and are directly related to insulin resistance which, in turn, plays a pivotal role in the pathogenesis of MetS. Accordingly, the evidence of a positive correlation between Hcy and C-peptide in the whole study population makes it reasonable to suppose that different concentrations of circulating Hcy could account for the different degree of insulin resistance among patients with MetS. Although scientific reports on this topic are not conclusive, our results are in line with previous observations showing that high serum levels of Hcy are associated with an increased risk to develop Type II diabetes mellitus, are closely associated to β-cell dysfunction and insulin resistance via oxidative- and inflammation-mediated pathways [28, 29] and may contribute to cause diabetes-related complications such as endothelial and cardiovascular damage [19, 30], retinopathy and nephropathy [20, 30–34]. The potential capacity of Hcy to mediate kidney damage is consistent with our findings of significant differences between the two groups of MetS patients with reference to the main renal function test parameters. This view is confirmed by the negative correlation between Hcy and e-GFR as well as by the positive correlation of Hcy with both BUN and creatinine noted in all the patients included in the study. However, renal function of MetS patients with high Hcy appeared to be preserved, suggesting that Hcy should rather be considered as an early marker of renal impairment in MetS, similarly to what was recently suggested [35].

As already described in the general population, we confirmed in patients with MetS the negative relationship between homocysteinemia and folic acid. Considering the key role of folates in the metabolism of methionine [12] and our observation of a significant reduction of folate concentrations in MetS patients with high Hcy, we suggest that any folic acid deficiency occurring in these patients be promptly corrected. Such a therapy would, in fact, improve this methionine metabolic disorder thought to contribute to the CV complications of the MetS, without any significant side effect. Vitamins B supplementation is undoubtedly effective in lowering serum Hcy levels, but data on its effects on CVR reduction are not conclusive. Evidence has been provided that Hcy-lowering vitamin B treatments failed to improve CV outcomes and to reduce death from any cause or stroke recurrence in patients with or without previous ischemic stroke or known CV disease, and this would not seem to recommend the systematic use of such therapeutic regimens in patients with a CV disease history [25, 36]. Conversely, it has also been reported that supplementation with folic acid, alone or in combination with vitamin B6 and B12, is effective in reducing stroke risk in patients with known CV disease [25, 37, 38] and, above all, can significantly decrease the risk for first stroke in hypertensive adults without a CV disease history [39–42]. This particular capacity of Hcy-lowering therapy to provide an effective primary prevention against CV events strengthens our proposal to systematically treat folic acid deficiency in MetS patients. Further support of this approach is provided by the outcomes of a recent systematic review and meta-analysis of several randomized controlled trials addressing the effects of metformin, a first-line insulin-sensitizing drug, on Hcy serum levels [43]. Authors concluded that, although metformin does not directly affect Hcy levels, it would induce an increase in the amino acid concentration when patients are not appropriately supplemented with group B vitamins and/or folates. This effect is considered evidently detrimental by the American Association of Clinical Endocrinologists, whose guidelines do, in fact, suggest that vitamin B12/folates be added in patients under treatment with metformin [44].

It cannot be denied that the real significance of high circulating Hcy has not yet been fully elucidated. To gain insight into this issue, we investigated the atherogenic role of Hcy in our insulin resistant patients. In this context, it should be emphasized that the group with high Hcy exhibited higher values of both IMT and carotid stenosis than the group with normal Hcy. Furthermore, in all patients with MetS, the increased Hcy concentration was paralleled by an analogous increase in IMT or carotid stenosis values. These findings are even more impressive considering that Hcy effects were not mediated by the smoking habit variable, being significant in non-smokers patients as well, nor by other well-known and potentially confounding atherosclerosis risk factors.

This allows us to speculate that Hcy may exert a direct role in the pathogenesis of vascular complications of MetS. Another intriguing aspect is that, even if uric acid shares with Hcy the ability to induce atherosclerosis-associated events (i.e. oxidative damage, endothelial cell dysfunction and smooth muscle cell proliferation), in our patients it did not exhibit any significant relationship with IMT or carotid stenosis, thus questioning its clinical role in the development of atherosclerotic process in the course of MetS.

Overall, the study has some potential limitations: (i) the number of patients would need to be increased in order to allow for some definitive conclusions to be drawn; (ii) although no patient used nutritional supplements, particular eating habits, potentially interfering with Hcy levels, were not directly evaluated.

Beyond these limitations, our results outline a strict relationship among insulin resistance, hyperhomocysteinemia and MetS, whose clinical and pathogenic interactions warrant further investigation. In addition, the enhancing role of Hcy on the progression of atherosclerotic process suggests that a correction of folate deficiency is advisable in order to prevent the CV morbidity and mortality that is so common in insulin resistant MetS patients.

## Data Availability

The datasets used during the current study are available from the corresponding author on reasonable request.

## Abbreviations

MetS: metabolic syndrome
Hcy: homocysteine
IMT: Intima Media Thickness
CV: cardiovascular
CVR: cardiovascular risk
HDL: high density lipoprotein
LDL: low density lipoprotein
BMI: Body Mass Index
HbA1c: glycated haemoglobin
NASCET: North American Symptomatic Carotid Endarterectomy Trial
HOMA: Homeostasis Model Assessment
SD: standard deviation
SE: standard error
e-GFR: glomerular filtration rate
BUN: blood urea nitrogen
SBP: systolic blood pressure
